# CKR-L3, a deletion version CCR6-isoform shows coreceptor-activity for limited human and simian immunodeficiency viruses

**DOI:** 10.1186/1471-2334-14-354

**Published:** 2014-07-01

**Authors:** Salequl Islam, Katsuaki Kanbe, Nobuaki Shimizu, Takahiro Ohtsuki, Atsushi Jinno-Oue, Atsushi Tanaka, Hiroo Hoshino

**Affiliations:** 1 Department of Virology and Preventive Medicine, Gunma University Graduate School of Medicine, Maebashi, Gunma 371-8511, Japan; 2 Department of Microbiology, Jahangirnagar University, Savar, Dhaka 1342, Bangladesh; 3 Department of Orthopaedic Surgery, Tokyo Women’s Medical University, Tokyo 116-8567, Japan; 4 Department of Microbiology and Cell Biology, Tokyo Metropolitan Institute of Medical Science, Tokyo 156-8506, Japan; 5 Research Institute for Microbial Diseases, Osaka University, Osaka 565-0871, Japan; 6 Department of Epidemiology, Johns Hopkins School of Public Health, Baltimore, MD 21205, USA

**Keywords:** HIV/SIV, Coreceptors, Chemokine receptors (CKRs), G protein-coupled receptors (GPCRs), CCR6 and CKR-L3

## Abstract

**Background:**

The chemokine receptors (CKRs), mainly CCR5 and CXCR4 function as major coreceptors in infections caused by human immunodeficiency virus (HIV) and simian immunodeficiency virus (SIV). Approximately 20 G protein-coupled receptors (GPCRs) have been identified as minor coreceptors, alike CCR6 that we reported recently. Since CKR-L3 is indentified as a natural isoform of CCR6, we attempted in this study to explore the coreceptor function of CKR-L3.

**Methods:**

NP-2 cells transduced with CD4-receptor (NP-2/CD4) normally remain resistant to HIV or SIV infection. However, the introduction of functional coreceptors can make these cells susceptible to these viruses. NP-2/CD4/CKR-L3 cells were produced to examine the coreceptor activity of CKR-L3. Likely, CCR6-isoform and the major coreceptors, CCR5 and CXCR4 were also examined in parallel. Presence of viral antigen in infected NP-2/CD4/coreceptor cells was detected by indirect immunofluorescence assay (IFA). The results were validated by detection of syncytia, proviral DNA and by measuring reverse transcriptase (RT) activities.

**Results:**

HIV-2MIR and SIVsmE660 were found to infect NP-2/CD4/CKR-L3 cells, indicative of the coreceptor function of CKR-L3. Viral antigens appeared faster in NP-2/CD4/CKR-L3 cells than in NP-2/CD4/CCR6, indicating that CKR-L3 is a more efficient coreceptor. Moreover, syncytia formation was more rapid and RT release evidenced earlier and at higher levels with CKR-L3 than with CCR6. Sequence analysis in the C2-V3 envelope region of HIV-2MIR replicated through CKR-L3 and CCR6 coreceptor showed two and three amino acid substitutions respectively, in the C2 region compared to the CCR5-variant. The SIVsmE660-CKRL3 variant showed three amino acid substitutions in the V1 region, one change in the V2 and two changes in the C2 region. The SIVsmE660-CCR6 variant produced two changes in the V1 region, and three in the C2 region.

**Conclusions:**

Isoform CKR-L3 exhibited coreceptor activity for limited primary HIV and SIV isolates with better efficiency than the parent CCR6-isoform. Amino acid substitutions in the envelope region of these viruses may confer selective pressure towards CKR-L3-use. CKR-L3 with other minor coreceptors may contribute to HIV and SIV pathogenesis including dissemination, trafficking and latency especially when major coreceptors become compromised. However, further works will be required to determine its clinical significance in HIV and SIV infection.

## Background

Human immunodeficiency virus (HIV) and simian immunodeficiency virus (SIV) infect susceptible cells by the interaction of the viral envelope (Env) glycoprotein gp120 with the CD4 receptor [1] and a suitable coreceptor, preferably one of the two chemokine receptors (CKRs), CCR5 or CXCR4 [2]. Some HIV-1, HIV-2 and SIV strains and isolates have the ability to use coreceptors independent of CD4 to infect target cells both *in vitro* and *in vivo*[3-5]. The coreceptor tropism is defined based on the ability of viruses to use particular coreceptors, hence dividing HIV and SIV strains into three primary groups: R5 viruses that use CCR5 as their coreceptor, X4 viruses that utilize CXCR4 as their coreceptor and R5X4 or dual tropic viruses that can use either of the two coreceptors [6]. The majority of viral strains initiate infection through the CCR5 receptor [7], then subsequently change their coreceptor shifting to CXCR4, probably expand to more other coreceptors [8]. The switching from R5 to X4 or multi-tropism accelerates CD4+ T cell depletion and rapid disease progression *in vivo*[9]. Apart from the major two coreceptors, more than 12 CKRs and several orphan G protein-coupled receptors (GPCRs) have been identified as coreceptors for different HIV and SIV strains [10,11]. Additional coreceptors using solely by SIV have also been demonstrated *in vivo* in Sooty mangabey infections in the CCR5-depleted condition [12]. Recently, we reported the role of CCR6, a 374 amino acid polypeptide, as a functional coreceptor for limited HIV and SIV isolates [13]. The open reading frame (ORF) of CCR6 carries a second exon-frame that initiates translation from the second methionine of the amino-terminal region (NTR) and encodes a polypeptide of 369 amino acids [14]. This alternate version of CCR6-isoform is known as CKR-L3 [15]. In this study, we investigated the coreceptor activity of CKR-L3 in comparison to CCR6. We find coreceptor activity of CKR-L3 to be stronger than that of CCR6 for the primary isolates, HIV-2MIR and SIVsmE660.

## Methods

### Cells

The human glioma cell line NP-2 expresses neither CD4 nor coreceptors, thus serving as an ideal host for the study of coreceptor activity [16]. Eagle’s minimum essential medium (EMEM) (NISSUI Co., Inc. Tokyo, Japan) supplemented with 10% fetal bovine serum (FBS) was used to maintain NP-2 cells and its derivatives. A panel of established human cell lines was examined for the expression of mRNA of CKR-L3- and CCR6-ORFs. T-cell lines, ATL-1 K [17], C8166, Jurkat [18] and a B-cell line, Daudi [19] were cultured in RPMI 1640 medium (NISSUI Co., Inc.) containing 10% FBS. Hepatoma cell lines, huH1 [20] and HepG2 [21] were cultured in Dulbecco’s modified EMEM (DMEM) (NISSUI Co., Inc.) containing 10% FBS. Human peripheral blood mononuclear cells (PBMC) were isolated from the blood of a healthy donor using Ficoll-Paque density gradient centrifugation (Pharmacia, Uppsala, Sweden), then stimulated with phytohemagglutinin (PHA) and cultured in RPMI 1640 medium supplemented with 20% FBS and 100 IU/ml of recombinant interleukin-2 (IL-2). A human T-cell line, C8166 was transduced with CCR5 to prepare C8166/CCR5 cells, which were used for the preparation of viral stocks of HIV and SIV strains [16]. The amphotropic packaging cell line Phoenix-Ampho [22] was maintained in DMEM containing 10% FBS.

### Viruses

A panel of laboratory-adapted HIV-1 strains, GUN-1WT, GUN-1V, GUN-4WT, GUN-4V, IIIB, BaL; and HIV-2 strains, CBL23, ROD, SBL6669, and SIV strains, mac251 and mndGB-1, were used to examine coreceptor activities [13]. Primary isolates of HIV and SIV were obtained from the National Institute for Biological Standard and Control (NIBSC, London, UK). The characteristics of these isolates with NIBSC-reference codes are as follows: HIV-1 strains, MVP-5180 (Cameroon, subtype O, EVA167), 93BR020 (Brazil, subtype F, ARP179.25), 92US723 (USA, subtype B, ARP1039.3), HAN2 (Germany, subtype B, EVA158). An HIV-2 strain, MIR (Guinia Bissau, EVA171), and an SIV primary isolate, smE660 (ARP1040), originated from a sooty mangabey, were also included. A primary isolate GUN11 was isolated from Gunma University Hospital and used in this study.

### Amplification of CKR-L3 open reading frame

The coding region DNA sequence of CKR-L3 was obtained from the GenBank database (Accession number, Z79784.1). Oligonucleotide primers were designed and synthesized (Proligo K.K., Tokyo, Japan) covering its ORF and to detect reverse-transcribed mRNA by PCR. Primers sequences, orientations and positions in the respective ORF were as follows: CKR-L3-F, 5′ ATGAATTTCAGCGATGTTTTCGACTCCAG 3′(sense: from the 1^st^ to the 29^th^) and CKR-L3-R, 5′ TCACATAGTGAAGGACGACGCATTGTCG 3′(antisense: 1083^rd^-1110^th^). The native CCR6-ORF (GenBank accession number, U60000.1) was amplified accordingly. As controls, the mRNA expression of CD4 (M12807.1), CCR5 (U54994.1), CXCR4 (AY242129.1) and gleceraldehyde-3-phospahte dehydrogenase (GAPDH, M17851.1) were also examined. Candidate ORFs amplified by RT-PCR were detected by electrophoresis through 1% (w/v) agarose gel.

### Cloning of CKR-L3 as coreceptor candidates

Amplified CKR-L3-ORF was cloned into a TA-cloning plasmid, pGEM-T Easy (Promega, Madison, WI), and the derivative plasmid was designated as pGEM-T Easy/CKR-L3. Sequencing of cloned DNA fragment was performed by a 5500-sequencer (Hitachi, Tokyo, Japan) using fluorescent primers labeled with Texas Red. Restriction-digested CKR-L3 ORF was re-cloned into an expression plasmid, pMX-puro [23] and transfected into NP-2/CD4 cells to produce and establish NP-2/CD4/CKR-L3 indicator cell line as described earlier [16]. Similarly, NP-2/CD4/CCR6 cell line was used as running control, NP-2/CD4/CCR5 and NP-2/CD4/CXCR4 as positive controls and NP-2/CD4 was used as a negative-control.

### HIV and SIV infection assay

Receptor and coreceptor transduced NP-2/CD4/CKR-L3, NP-2/CD4/CCR6, NP-2/CD4/CCR5, NP-2/CD4/CXCR4 and NP-2/CD4 cells were seeded into 24-well microtiter plates in a density of 50,000 cells/mL. After overnight, medium was drained off with the addition of viral inoculums to the seeded-cells. Following a six hour incubation, the cells were washed three times with EMEM containing 10% FBS to remove free virus, then refilled with 500 μL fresh medium per well and cultured at 37°C in a 5% CO_2_ incubator. In 3–5 day intervals, the cell monolayers were disrupted with trypsin-EDTA (0.25% trypsin, 1 mM EDTA), cell viability was checked by 0.4% trypan blue staining solution, counted via hemacytometer and re-seeded equal number of cells to maintain uniform cell growth rate and maintained for up to 8 weeks. The presence of viral antigens in the infected cells was detected by indirect immunofluorescence assay (IFA) [24]. Retroviral infection was further assured by detection of proviral DNA by PCR using the genomic DNA of infected cells as templates [25]. Infection induced cell fusion was observed under light microscopy. Formation of syncytia was detected by Giemsa staining (Muto Pure Chemicals, Tokyo, Japan). Subsequently, virus releases through different coreceptors were quantified with measurement of reverse transcriptase (RT) activities in used culture supernatants [17]. Briefly, culture supernatants of virally infected cells were precipitated with 30% polyethylene glycol and lysed with standard disruption buffer containing 0.026% triton X-100. Lysates were mixed with 50 μL of assay reaction mixture consisting of 40 mM Tris HCl (pH 7.8), 4 mM dithiothreitol, 45 mM KCl, poly(rA), oligo(dT) (Pharmacia Biotech, NJ), 15 μM [^3^H] (5 Ci/mmol) (Amersham International, UK), and 10 mM MgCl_2_. The mixtures were incubated for one hour at 37°C. Incorporation of radioactivity into cold trichloroacetic acid–insoluble fractions was assayed in a liquid scintillation counter (Beckman Coulter LS 6500, CA). Ionization rate in counts per minute (cpm) of culture supernatants was adjusted by subtracting the background cpm. The assay was carried out in duplicates.

### Sequencing of viral DNA

The primer pair for proviral DNA of HIV-2MIR was HIV2-F (forward 5′-ATGTGATAAGCACTATTGGGATGATA-3′) and HIV2-R (reverse 5′-CATGCTTGTTTAGGTCTTGTATTGAT-3′), located between positions of 7084-7109-nt and 7433-7458-nt, respectively, covering the C2-V3 *env* region (nucleotide positions of HIV-2CRIK, DQ307022.1). For SIVsmE660, a proviral DNA fragment of 773 nucleotides spanning the V1-V3 regions of *env* gene was amplified as described earlier [26]. Upon detection, the DNA bands were cloned into a TA-cloning plasmid, pGEM-T Easy, and inserts were sequenced and blasted in the NCBI nucleotide database (http://blast.ncbi.nlm.nih.gov) to confirm the DNA integrity of the inoculated viruses. Sequence alignments have been done using the BioEdit program (version 7.2) for windows.

### Statistical analysis

Mean percentages of the antigen-positive cells in two independent infection assay and RT values in duplicate samples were analyzed by *paired t* test using IBM SPSS statistics data editor (version 17).

### Nucleotide sequence accession numbers

The viral nucleotide sequences used in this study were submitted to GenBank and were assigned accession numbers: JN107567-69 for HIV-2MIR-CCR5, HIV-2MIR-CKR-L3 and HIV-2MIR-CCR6, and the number JN107564-66 for SIVsmE660-CCR5, SIVsmE660-CKR-L3, SIVsmE660-CCR6 clones, respectively.

### Ethics statement

This study was approved by the ethical review committee of the Faculty of Medicine, Gunma University, Japan, and standard written consent was obtained from the enrolled healthy blood donor.

## Results

### Detection of CKR-L3 and CCR6 expressions

**Figure 1:**
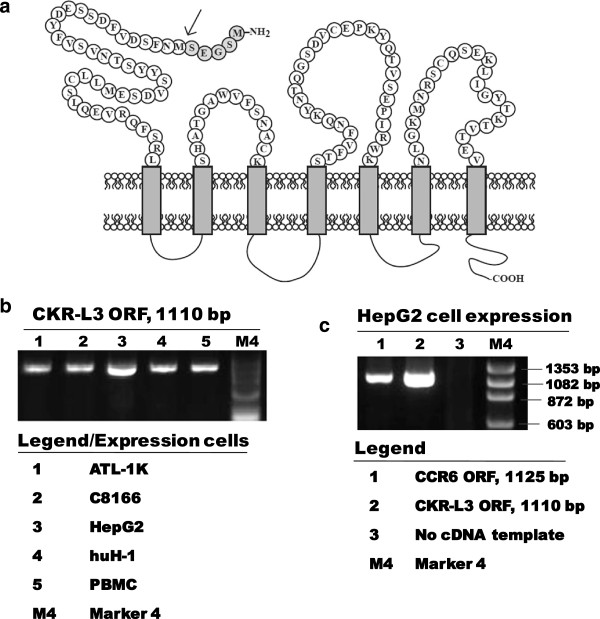
**Schematic structures of CKR-L3, CCR6 and their expression in different human cell lines. a)** The amino acid sequence of NTR of CCR6 isoform; shaded residues indicating region absent in CKR-L3 isoform. The arrow indicates the starting point for the translation for CKR-L3. **b)** The expression of CKR-L3 in different human cells was detected by reverse transcriptase-PCR. The products shown in column 1 to 5 indicate the mature full-length open reading frame (ORF) of CKR-L3 and right-most column shows molecular weight of marker. The cell lines expressed CKR-L3-cDNA are specified separately in legend below. **c)** Comparative ORFs of CCR6 and CKR-L3 expressed in HepG2 cells.

The CKR-L3 ORF is similar to that of CCR6 except for the absence of first five-amino acids in the NTR region. The schematic diagram in Figure 1a indicates the point of deletion at native CCR6 to represent CKR-L3. Expressions of mRNA of both CKR-L3 and CCR6 were detected by RT-PCR in PBMC, T cell lines, ATL1K and C8166 as well as in hepatic cell lines, such as huH1 and HepG2 (Figure 1b). Similar to our findings, previous reports have showed the expression of CKR-L3 in spleen, lymph nodes and PBMC [15], whereas CCR6 was selectively expressed on Th17 cells and regulatory T cells, memory T cells, B cells and dendritic cells [27,28]. The 15-nucleotide size-difference between the amplified ORFs of CKR-L3 and CCR6 could not be distinguished fairly when they were run through 1.5% agarose gel electrophoresis (Figure 1c). The major coreceptor, CCR5 was found to be expressed on ATL-1 K and PBMC. The expression of CD4 was detected on both the T cell lines and on PBMC.

### Establishment of NP-2/CD4/coreceptor cells

Amplified ORFs of CKR-L3, CCR6 and CCR5 were transduced one by one into NP-2/CD4 cells to produce NP-2/CD4/coreceptor cells. The expression of the coreceptor candidates were affirmed by detecting their specific mRNAs in the transfected cell lines by RT-PCR. We ensured the expressions of respective candidates in NP-2/CD4/coreceptor sub-lines by flow cytometry (FCM) following previously described methods [29]. The mother cell, NP-2/CD4 did not express any of the coreceptor candidates.

### Coreceptor functions of CKR-L3 and CCR6

**Figure 2:**
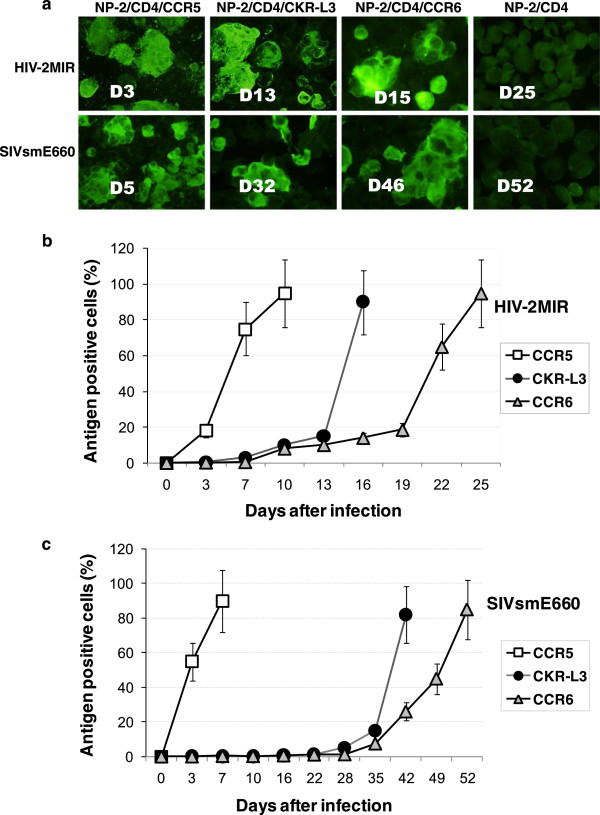
**Immunofluorescence assay to detect the susceptibilities of NP-2/CD4/coreceptor cells and infection dynamics of HIV-2 and SIV through different coreceptors. a)** NP-2/CD4/coreceptor cells were seeded and next day inoculated with primary isolates, HIV-2MIR and SIVsmE660. Cells were passaged in 3–4 day intervals and continued for four weeks for HIV-2 and eight weeks for SIV. Viral antigens in infected cells were determined by IFA, for which cells were smeared onto glass slides in duplicate, dried, fixed with acetone and stained with pooled human/monkey anti-HIV/SIV sera for HIV-2MIR- and SIVsmE660-antigen, respectively. Fluorescein isothiocyanate (FITC)-conjugated goat anti-human and anti-monkey IgG were used as secondary antibodies. Specific days after inoculation on infected cells were shown accordingly. **b)** Mean percentages of the antigen-positive cells in the two independent infection assay was calculated using IBM SPSS statistics data editor. HIV-2MIR started progressive replication via CKR-L3 and CCR6; less than 10% cells became infected within the first week after inoculation and gradually more than 80% cells became antigen-positive at day-16 and day-25 after inoculation, respectively. **c)** SIVsmE660 started slow infection and took more adaptation time using CKR-L3 and CCR6 as coreceptors. The virus infected less than 10% of cells with in the first four weeks of inoculation via either of the coreceptors, however, showed progressive infection to make more than 80% cells become antigen-positive at six weeks via CKR-L3 and almost eight weeks via CCR6.

The coreceptor activities of CKR-L3 and CCR6 for HIV-2MIR and SIVsmE660 primary isolates were noticed when viral antigen-positive NP-2/CD4/CKR-L3 and NP-2/CD4/CCR6 cells were detected by IFA (Figure 2a). We have described the coreceptor-role of CCR6 in detail previously [13]. The present report highlights the superior coreceptor-role of CKR-L3 relative to its native counterpart, CCR6. HIV-2MIR showed a good pace of replication in NP-2/CD4/CKR-L3 cells and more than 80% cells became antigen positive within 16 days after inoculation (Figure 2b). To reach the same level of infection, CCR6-transfected cells took one week more. In contrast, HIV-2MIR attained the same level of infection in three to four days in CCR5-transfected cells (Figure 2b). SIVsmE660 isolate was also highly infective via the CCR5 coreceptor: more than 80% NP-2/CD4/CCR5 cells were antigen-positive within one week after inoculation. This virus was slow but progressive through the CKR-L3 coreceptor and took six weeks to infect 80% of its cells. In contrast, CCR6 showed much weaker coreceptor activity than CKR-L3 taking 10 days more to produce similar levels of infection (Figure 2c). However, infections via another major coreceptor, CXCR4, is not shown, but described in Table 1. A second independent experiment had been done that revealed similar outcomes.

**Table 1:** Replication efficiency of laboratory-adapted strains and primary isolates via different coreceptors in NP-2/CD4/coreceptor cell line system

**Virus**		**Coreceptor in NP-2/CD4 cells:**				
		**CCR5**	**CXCR4**	**CKR-L3**	**CCR6**	**None**
**Laboratory isolates**						
HIV-1	IIIB	-	++++^a^	-	-	-
	BaL	++++	-	-	-	-
	GUN1wt	++++	++++	-	-	-
	GUN1v	++	+++	-	-	-
	GUN4wt	+++	+++	-	-	-
	GUN4v	++	+++	-	-	-
HIV-2	CBL23	++	++++	-	-	-
	ROD	++	++++	-	-	-
	SBL6669	-	++++	-	-	-
SIV	Mac251	++++	-	-	-	-
	GBmnd1	++++	++	-	-	-
**Primary isolates**						
HIV-1	MVP5180	++++	++++	-	-	-
	93BR020	++++	+++	-	-	-
	92US723	++++	-	-	-	-
	HAN2	++++	++++	+	+	-
	GUN11	++++	++++	-	-	-
HIV-2	MIR	++++	++++	+++	+++	-
SIV	smE660	++++	-	+++	++	-

^a^Viral antigen positive cells were examined by IFA.

++++,70-90% of cells became antigen-positive within one week after infection; +++,70-90% of cells became antigen-positive within two to four weeks of infection; ++,70-90% of cells became antigen-positive within five to eight weeks of infection; +,1-2% of cells become infected initially, however, did not progress with time untill eight weeks of infection and -,<0.1% of cells became antigen-positive within seven to eight weeks of infection.

**Figure 3:**
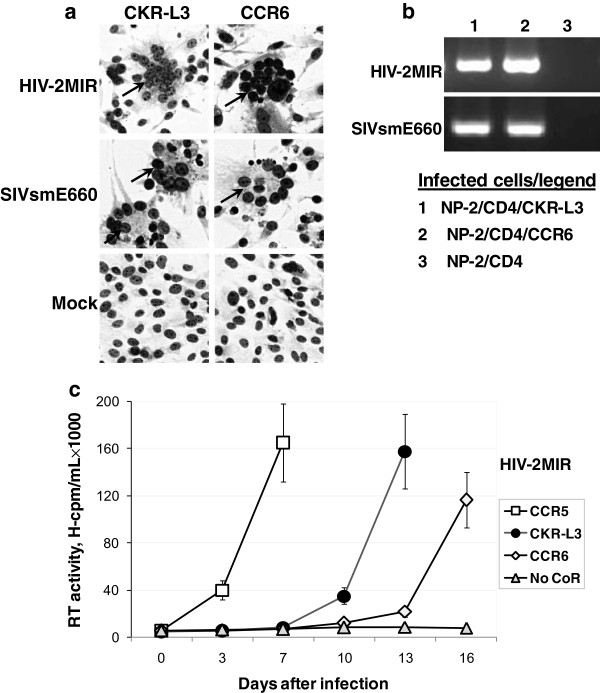
**Retrovirus-induced syncytia formation, proviral DNA detection and RT assay. a)** Syncytia formation of HIV/SIV as a sign of acute infection was manifested by nuclear aggregation in multinucleated giant cells (MGC) detected by Giemsa staining. Both HIV-2MIR and SIVsmE660 induced syncytia formation in NP-2/CD4/CKR-L3 and NP-2/CD4/CCR6 cells. However, syncytia formation in the former cell line was shorter than the latter for both viral variants. **b)** As evidence of retroviral DNA integration into the host cell genome, proviral DNA was detected by PCR using the genomic DNA of the infected NP-2/CD4/CKR-L3 and NP-2/CD4/CCR6 cells as templates. **c)** Reverse transcriptase (RT) assay was done to detect HIV-2MIR replication efficiency using CKR-L3 and CCR6 as coreceptors. Culture supernatants of infected cells were harvested at different time points of infection-cycle. Mean cpm values of the duplicate samples were determined. CKR-L3 mediated increased and more rapid RT release than that of CCR6.

Productive HIV and SIV infection in susceptible cells *in vitro* or *in vivo* is often associated with the formation of syncytia, which are multinucleated giant cells (MGC) [30,31]. MGC in infected NP-2/CD4/CKR-L3 and NP-2/CD4/CCR6 cells were quantified after fixation and staining with Giemsa. Both HIV-2MIR and SIVsmE660 induced syncytia formation using CKR-L3 and CCR6 as coreceptors (Figure 3a). However, syncytia formation through the CKR-L3 coreceptor occurred more rapidly than with the CCR6 coreceptor. Clusters were variable in sizes and consisting of 10–25 nuclei per syncytium. Infection through CCR5 frequently generated viral-induced MGC, while coreceptor-negative NP-2/CD4 cells did not form any cluster of nuclei (data not shown). Typically when cells become infected by HIV and SIV, viral DNA provirus forms by reverse transcription of genomic RNA into double stranded DNA followed by subsequent integration into host DNA [32]. We further demonstrated infection through the CKR-L3 and CCR6 coreceptors through the detection of proviral DNA in infected cell lines (Figure 3b). Retroviral cytopathic effects (CPE) in cell-line-systems release a large amount of virus in to cell-supernatant. The amount of produced viruses was assessed by RT activities in spent culture media of viral inoculated cells [33]. We observed sharp increases of RT activities associated with CPE and cell ruptures. For HIV-2MIR infection, RT release was measured at different time points with a time dependent gradual increase of antigen-positive cells as detected by IFA (Figure 3c). The RT activities at the final time point of infection cycles were 1.7 × 10^5^, 1.6 × 10^5^ and 1.2 × 10^5^ cpm/mL using CCR5, CKR-L3 and CCR6 coreceptors respectively when 80-90% cells become antigen positive by IFA. All the three cell lines generated four to six fold increases of RT values during the last three days of respective infections (Figure 3c). However, the CKR-L3-transfected cell line took a shorter time to release peak RT values compared to that of CCR6. Contrast to HIV-2MIR, RT activities for SIVsmE660 infections were measured only at the end points of each infection cycles. Culture fluids of infected NP-2/CD4/CCR5 cells on day-seven, NP-2/CD4/CKR-L3 cells on day-42 and NP-2/CD4/CCR6 cells on day-52 were assayed for RT activities when CPE ascended at the level of 80-90% cell infection (Figure 2c). The RT releases using CCR5, CKR-L3 and CCR6 coreceptors for SIVsmE660 were 2.3 × 10^6^, 1.7 × 10^6^ and 1.5 × 10^6^ cpm/mL, respectively. Very negligible RT activities (0.8-1.5 × 10^3^) were detected when the supernatants of virus-infected NP-2/CD4 cell-cultures were measured.

### Sequence analyses of the proviral DNAs

**Figure 4:**
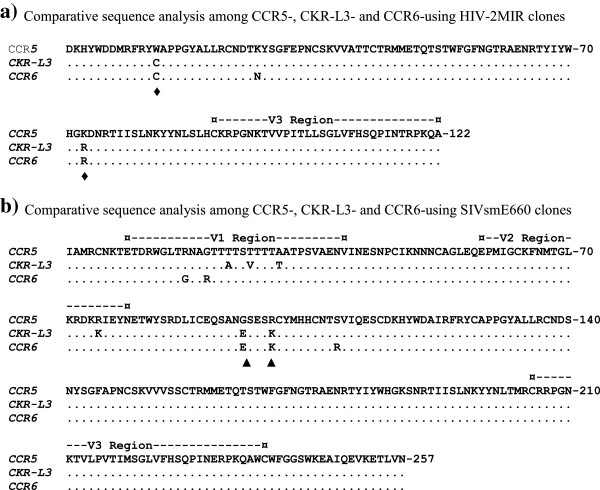
**Sequence analysis of CKR-L3- and CCR6-variant of HIV-2MIR and SIVsmE660 isolates.** The alignment of the C2-V3 amino acid sequences of the HIV-2MIR for CKR-L3- and CCR6-variant has been made with the parental CCR5-variant. Similarly, alignment was made of the V1-V3 amino acid sequences of the SIVsmE660 for the three variants. **a)** Each clone of CCR5-, CKR-L3- and CCR6-variant of HIV-2MIR has been aligned. Dots indicate identity and letters represent substitutions in the adapted variants relative to the parental isolate. The positions of the common amino acid changes are marked by ♦. The V3 domain is marked by dashes. **b)** Amino acid sequences of the three clones each from CCR5-, CKR-L3- and CCR6-variant of SIVsmE660 covering the V1-V3 domains were aligned. The regions equivalent to V1, V2 and V3 are indicated by dashes. Dots indicate the identity with the parental isolate; letters represent differences in the adapted variants. The positions of common amino acid changes are marked by ▲.

The sequences of HIV-2MIR and SIVsmE660 grown in NP-2/CD4/CCR5, NP-2/CD4/CKR-L3 and NP-2/CD4/CCR6 cells were validated by blasting with NCBI and GenBank nucleotide databases. To analyze the genetic divergence while propagating through different coreceptors, we aligned CCR5-clone to those of CKR-L3- and CCR6-clones. For HIV-2MIR, the amplified C2-V3 region showed good integrity in the majority of the nucleotide sequences among three clones. However, four common nucleotide substitutions were found in the V3 region and three common changes were found in the C2 region of the CKR-L3- and CCR6-clones compared to the CCR5-clone sequence (Additional file 1). All of the V3-substitutions were synonymous, revealing no amino acid change. Unlike the V3 region, two non-synonymous substitutions, namely lysine (K) to arginine (R) and tryptophan (W) to cysteine (C) were observed in the C2 region. Another substitution of lysine to asparagine (N) was found in the CCR6-clone, but not in the CKR-L3-using isolate (Figure 4a).

The *env* gene for the V1-V3 region of CKR-L3- and CCR6-variants of SIVsmE660 exhibited remarkable nucleotide substitutions relative to that of the parental CCR5-smE660: the levels were 1.4% (11/773) in smE660-CKR-L3 and 1.2% (9/773) in smE660-CCR6 clones. Of these four substitutions were common for both clones (Additional file 2). Some of the nucleotide changes were synonymous. However, non-synonymous substitutions of the CKR-L3-variant were observed in the V1 region, with the change of two threonines (T) into alanine (A) and valine (V) and one alanine (A) into threonine (T). On the other hand, the CCR6-variant demonstrated two changes, arginine (R) to glycine (G) and vice-versa in the V1 region (Figure 4b). In the V2 region, the CKR-L3-clone showed one amino acid change, arginine (R) to lysine (K). The CCR-6 clone remained unaltered. No amino acid substitution was found in the V3 region of either the CKR-L3 or CCR6 clones. Interestingly, the C2 region showed two amino acid changes in the CKR-L3 variant, glycine (G) to glutamic acid (E) and arginine (R) to lysine (K). Likely, the CCR6-variant also showed changes of glycine (G) to glutamic acid (E) and arginine (R) to lysine (K) and in addition one serine (S) to arginine (R) (Figure 4b).

## Discussion

The majority of prior studies focused on coreceptor usage by HIV and SIV have been concentrated on the two major chemokine receptors, CCR5 and CXCR4. But many HIV and SIV isolates have been reported to use alternative coreceptors in *in vitro* assays, belonging to CKRs and the orphan receptors such as CCR3 [34], CCR8 [35], FPRL1 [36], GPR15 [37] and several others. There are many hurdles in demonstrating the contribution of these alternative coreceptors to viral entry *in vitro* and their clinical relevance *in vivo*. Recent *in vivo* studies have showed that SIV infects CCR5-null cells, evidencing the physiologic relevance of alternative coreceptors [12,38]. SIV strains were found to competently infect cells utilizing multiple GPCRs, of both human and sooty mangabey (SM)-origin as their entry cofactor. However, CCR6 remains the latest member of the coreceptor family that we have reported recently [13]. Like many other GPCRs, the CCR6 has a natural isoform named CKR-L3. Contrarily, in this study, we observed that CKR-L3 exhibited greater coreceptor-activity than the primary CCR6 counterpart. Database (UniProtKB/Swis-Prot) shows that CCR6 and CKR-L3 are encoded in the same genome and expected to be translated from the first and second methionines, respectively. Similarly, expression of isoforms of the major coreceptor, CXCR4 were detected earlier and their respective functions in chemokine signaling and coreceptor activities have been described previously [39,40]. However, there has been no reported isoform of the major coreceptor CCR5 so far.

Initial HIV infection is considered to be facilitated via CCR5 coreceptor activity [8] with evolution with CXCR4 during the later stage of infection [41]. However, a recent data has demonstrated CXCR4-tropic viruses in early-diagnosed infection, increasing the possibility of using CXCR4 in early viral transmission [42]. So, together with our findings, these data reflect the complexity of coreceptor shifting during HIV infection and the putative wider spectrum of coreceptor activity during such infections.

In this study, we found that CKR-L3 facilitated infection by three out of 18 HIV and SIV isolates tested. CCR6 also facilitated infection by an equal number of viruses in our previous study yielded. In contrast, both CCR5 and CXCR4 supported infection by the majority of isolates tested (Table 1). These findings suggest CCR6 and CKR-L3 are less-efficient coreceptors than the primary coreceptors, CCR5 and CXCR4. However, these minor coreceptors might be important entry pathways for viral infection when major coreceptors are compromised or blocked as described earlier [38].

CKR-L3 is a five amino acid deletion mutant from NTR compared to CCR6 providing accelerated and better infection efficiency in the NP-2/CD4 cell system. The findings of our studies imply that the first five amino acids in CCR6 are not essential for its coreceptor functioning, which rather carry plausible negative roles in coreceptor function. In a nutshell, the shorter coreceptor version carries better coreceptor function. Similar findings remain to that of CXCR4 isoforms as well [39,40]. On the other hand, among the deleted portion of CKR-L3, there was no tyrosine residues, which were reported to be part of the NTR of CCR5 considered vital for viral entry [43]. Potential functional roles of tyrosines in the CXCR4-NTR has been described in our previous reports, where we showed a partial deletion of CXCR4-NTR containing tyrosines abolishes its coreceptor function for many dual tropic HIV and SIV viruses [44]. Therefore, the natural deletion of a small NTR-part without tyrosine from CCR6 does not exert a deleterious coreceptor-function, rather promotes CKR-L3. We assume that the increased efficiency of CKR-L3 for viral entry is facilitated by the formation of a protein structure that is more amenable to interact with the retroviral envelope than that of the parent CCR6 protein.

We observed that sequence comparison of the CKR-L3- and CCR6-variants of HIV-2MIR to the CCR5-variant showed no divergence in the V3 region. However, two- and three-amino acid changes were found for CKR-L3- and CCR6-variants in the C2 region, respectively. These mutations in the C2 region may help the virus to adapt to new coreceptor usage. Further sequencing analysis covering the full *env* region may provide more detailed information of the possible mutations towards the evolution of new quasispecies.

Similar to HIV-2MIR, the V3 region of the recovered CKR-L3- and CCR6-variants of SIVsmE660 did not show any amino acid changes compared to the CCR5-isolate. These findings are congruent with a previous study that described the region corresponding to the V3 of HIV-1 as highly conserved in SIV [45] and the genetic variation of SIV as different from that of HIV-1 [46]. Our examination revealed the V1 and V2 regions of SIVsmE660 were not conserved as like the V3 region. These findings are also consistent with other reports describing sequence variation mostly in the V1 region of SIV infections [47,48]. Therefore, the V1 and V2 regions in the envelope glycoprotein may carry the potentiality in determining receptor tropism. Moreover, the CKR-L3-SIV variant showed more changes in the V1 and V2 regions compared to its CCR6-variant. These additional mutations may provide the recovered SIVsmE660 with better adaptability, leading to accelerated coreceptor activity for CKR-L3 as compared to CCR6. More detailed sequence analyses throughout the *env* gene or further full-length sequencing may provide more conclusive evidence for coreceptor-adaptation.

## Conclusions

Our studies demonstrating the role of CKR-L3, an isoform of CCR6, has extended our knowledge of viral pathogenesis. Further research will be needed to better understand how these minor coreceptors may contribute to disseminating viral infection *in vivo*. Through validated experiments, we have shown that the shorter isoform, CKR-L3 functioned as a better coreceptor than that of its parent CCR6. The molecular basis behind the variation of the coreceptor-strength between CKR-L3 and CCR6 requires further detailed investigation.

## Abbreviations

7TM: Seven-Transmembrane Receptor; C2: Constant region 2; CCR: CC chemokine receptor; CD4: Cluster of differentiation 4; CKRs: Chemokine Receptors; CPE: Cytopathic effects; DMEM: Dulbecco’s Modified EMEM; EMEM: Eagle’s Minimum Essential Medium; FBS: Fetal Bovine Serum; FCM: Flow cytometry; GAPDH: Gleceraldehyde-3-phospahte Dehydrogenase; GPCRs: G Protein-Coupled Receptors; HIV: Human Immunodeficiency Virus; IFA: Immunofluorescence Assay; IL: Interleukin; MGC: Multinucleated giant cells; NCBI: National Center for Biotechnology Information; NIBSC: National Institute for Biological Standard and Control; NTR: Amino Terminal Region; ORF: Open Reading Frame; PCR: Polymerase Chain Reaction; PBMC: Peripheral blood mononuclear cells; PHA: Phytohemagglutinin; SIV: Simian Immunodeficiency Virus; R5: Viruses that use CCR5; RPMI: Roswell Park Memorial Institute; RT: Reverse Transcriptase; V3: Variable region 3; X4: Viruses that use CXCR4.

## Competing interests

The authors declare that they have no competing interest.

## Authors’ contribution

SI conducted major laboratory experiments and virological assays, prepared the results and drafted the manuscript. KK designed the coreceptor candidate and cloned it into cells, NS guided various experiments and assisted with data acquisition, TO participated in virus preparation, AT worked on the establishment of cell lines, AJ assisted with microscopy and sequencing. HH played a vital role in reviewing and coordinating the study. All authors read and approved the final manuscript.

## Pre-publication history

The pre-publication history for this paper can be accessed here:

http://www.biomedcentral.com/1471-2334/14/354/prepub

## Supplementary Material

Additional file 1 **The nucleotide sequences of the C2-V3 domains of CCR5-, CKR-L3- and CCR6-clones of HIV-2MIR were aligned together.** Dots indicate the identity with the parental isolate; letters represent differences in the adapted variants. The V3 domain is indicated by dashes. Click here for file

Additional file 2 **The nucleotide sequences of CCR5-, CKR-L3- and CCR6-clones of SIVsmE660 covering V1-V3 regions were aligned.** Dots indicate the identity with the parental isolate; letters represent differences in the adapted variants. The regions equivalent to the V1, V2 and V3 of SIV are indicated by dashes. Click here for file
