# Supplementary material for: CKR-L3, a deletion version CCR6-isoform shows coreceptor-activity for limited human and simian immunodeficiency viruses
Source: BMC Infect Dis. 2014 Jul 1;14:354. doi: 10.1186/1471-2334-14-354 (PMC4089560; doi:10.1186/1471-2334-14-354)
Supplement: Additional file 1 — The nucleotide sequences of the C2-V3 domains of CCR5-, CKR-L3- and CCR6-clones of HIV-2MIR were aligned together. Dots indicate the identity with the parental isolate; letters represent differences in the adapted variants. The V3 domain is indicated by dashes. [file 1471-2334-14-354-S1.pdf]

Additional file 1: Alignment of the nucleotide sequences of the C2-V3 regions of HIV-2MIR lones propagated through CCR5, CKR-L3 and CCR6 coreceptors respectively

```
                 10        20        30        40        50        60        70
                  |         |         |         |         |         |         |
CCR5     gataagcactattgggatgatatgaggtttagatactgggcaccaccgggttatgccttgctaagatgca
CKR-L3   ......................................t...............................
CCR6     ......................................t...........g...................

CCR5     atgataccaagtattcaggctttgagcccaattgttctaaagtagtagctactacatgcactaggatgat-140
CKR-L3   .............................................................c........
CCR6     ..........t...........................................................

CCR5     ggaaacgcaaacttctacgtggtttggctttaatggcacaagagcagagaatagaacatatatctattgg-210
CKR-L3   ..................a...................................g...............
CCR6     ......................................................g...............

                                                                     ¤---------
CCR5     catggtaaagataacagaactatcatcagcttaaacaaatattataatctcagcttgcattgtaagaggc-280
CKR-L3   .......g..............................................................
CCR6     .......g..............................................................

         -----------------------------V3 Region--------------------------------
CCR5     caggaaataaaacagtggtgccaataacacttctttcaggattagtgtttcactcccagccaatcaatac-350
CKR-L3   .g.................a..............c..........................g........
CCR6     .g.................a..............c..........................g........

         ------------------
CCR5     aagacctaaacaagcatg-368
CKR-L3   ..................
CCR6     ..................
```
