# Supplementary material for: CKR-L3, a deletion version CCR6-isoform shows coreceptor-activity for limited human and simian immunodeficiency viruses
Source: BMC Infect Dis. 2014 Jul 1;14:354. doi: 10.1186/1471-2334-14-354 (PMC4089560; doi:10.1186/1471-2334-14-354)
Supplement: Additional file 2 — The nucleotide sequences of CCR5-, CKR-L3- and CCR6-clones of SIVsmE660 covering V1-V3 regions were aligned. Dots indicate the identity with the parental isolate; letters represent differences in the adapted variants. The regions equivalent to the V1, V2 and V3 of SIV are indicated by dashes. [file 1471-2334-14-354-S2.pdf]

Additional file 2: Alignment of the nucleotide sequences of the V1-V3 regions of SIV-smE660 clones propagated through CCR5, CKR-L3 and CCR6 coreceptors respectively

```
                10        20        30        40        50        60        70
                 |         |    ¤-------------V1 Region-----------------------
CCR5    attgcaatgagatgtaataaaactgagacagataggtggggtttgacaagaaacgcagggacaacaacaa
CKR-L3  ..................................................................g...
CCR6    ................................................g........a............

        ----------------------------------------------¤
CCR5    catcaacaacaacaacagcagcaacaccaagtgtggcagaaaatgttataaatgaaagtaatccttgtat-140
CKR-L3  .....gt..........a................a...................................
CCR6    ..................................a...................................

                                       ¤-------------V2 Region----------------
CCR5    aaaaaataataattgtgcaggcttggaacaggagcccatgataggttgtaaatttaacatgacagggtta-210
CKR-L3  ......................................................................
CCR6    ............c.........................................................

        -----------------------------¤
CCR5    aaaagggacaaaaggatagaatataatgaaacatggtattcaagagatttaatctgtgagcagtcagcga-280
CKR-L3  .............a........................................................
CCR6    ......................................................................

CCR5    atggaagtgagagtagatgttacatgcatcattgtaacaccagtgttattcaggaatcctgtgacaagca-350
CKR-L3  ...a...........a......................................................
CCR6    ...a...........a...........................a..........................

CCR5    ttattgggatgccattagatttagatattgtgcaccgccaggttatgctttgcttaggtgtaatgattca-420
CKR-L3  ............t.........................................................
CCR6    ............t.........................................................

CCR5    aattattcaggctttgctcctaactgttctaaggtagtggtttcttcatgcacaagaatgatggagacgc-490
CKR-L3  ..........................c.........................................a.
CCR6    ......................................................................

CCR5    aaacctctacttggtttggcttcaatggtactagggcagaaaatagaacatacatttattggcatggcaa-560
CKR-L3  ......................................................................
CCR6    ..................................a...................................

                                                            ¤-----------------
CCR5    aagtaatagaaccataattagcttaaataagtattataatctaacaatgagatgtagaagaccaggaaat-630
CKR-L3  ......................................................................
CCR6    ......................................................................

        -------------------------------- V3 Region ---------------------------
CCR5    aagacagttttaccagtcaccattatgtcagggttggtcttccattcgcaacccataaatgagagaccaa-700
CKR-L3  ......................................................................
CCR6    ......................................................................

        --------------
CCR5    aacaggcctggtgctggtttggaggaagctggaaagaggccatccaggaagtgaaggaaaccttggtcaa-770
CKR-L3  ......................................................................
CCR6    ......................................................................

CCR5    tca-773
CKR-L3  ...
CCR6    ...
```
